# A Decade-Long Comparative Study of Awareness and Knowledge of Oral Cancer Among Cypriots: Implications for Health Policy

**DOI:** 10.7759/cureus.93083

**Published:** 2025-09-24

**Authors:** Chrystala Charalambous, Aristomenis Syngelakis, George Pantelas, Maria Myrto Solomou

**Affiliations:** 1 School of Dentistry, European University Cyprus, Nicosia, CYP; 2 Oral and Maxillofacial Surgery, European University Cyprus, Nicosia, CYP; 3 Oral Medicine, European University Cyprus, Nicosia, CYP

**Keywords:** awareness, cyprus, oral cancer, oral health promotion, screening

## Abstract

Introduction: Oral cancer (OC) is a major health problem that is associated with high mortality and morbidity rates. Reducing diagnostic delay to achieve earlier detection is a cornerstone to improve survival and quality of life. Prevention and early detection are related to public awareness.

Aims: This study aimed to determine the awareness and knowledge of symptoms and signs of OC among Cypriots attending mobile dental clinics during an oral health campaign organized by the Public Dental Services (PDS) and the Clinic of The Oral and Maxillofacial Surgery of the Nicosia General Hospital (NGH) and compare them with the results of a similar study that was conducted in 2012.

Materials and methods: A cross-sectional study was conducted using an anonymous self-completed questionnaire, which was distributed to people visiting the mobile dental clinics in four major cities in Cyprus, during the preventive campaign for oral cancer, which took place in the first week of December 2021. Prior to their free oral examination and oral health advice, people were asked to fill out an anonymous questionnaire. The questionnaire included questions about socio-demographics, habits (smoking, alcohol consumption, visits to the dentists), plus knowledge and perceptions of OC. The results were compared with a similar study conducted in 2012 using the same questionnaire. The study was approved by the Bioethical Committee of Cyprus.

Results: One hundred and seventy-two people completed the questionnaire. 73.8% (n= 127) of the respondents stated that they have already heard about OC compared with 53% in 2012 (p<0.01). 85.4 % (n=147) identified smoking as a risk factor, 57% (n=98) alcohol consumption, and 33.7% (n= 58) solar irradiation. 27.3% (n=47) thought that OC is hereditary, compared with 38.9% in 2012. Although 58.7% (n= 101) mentioned that they had heard about screening for OC, only 19.8% (n= 34) reported that they had been screened for OC by their dentist; this percentage was higher compared with the equivalent in 2012, which was 12% (p<0.01). In addition, 36.6% (n= 63) reported that in case of a wound/lesion of their oral mucosa, they would seek treatment from a dentist compared with 25.6% in 2012 *(p*<0.05). In both studies, a higher level of education and frequent visits to the dentists were positively related to increased knowledge of OC signs and risk factors and were more likely to receive preventive screening (p<0.05).

Conclusions: Although the results show an improvement in the level of knowledge among Cypriots regarding OC, still disparities exist among different socioeconomic groups, highlighting the need for oral health promotion and an holistic and multisectoral approach, which will enable people taking the necessary preventive measures but also will encourage frequent visits to dentists for a dental examination and screening for OC in order suspicious lesions to be referred to maxillofacial surgeons.

## Introduction

Oral cancer (OC) remains a significant global public health issue, with a notably uneven distribution across regions due to varying exposure to modifiable risk factors. It is considered the 13th most prevalent malignancy in the world [1]. Globally, there were 188438 fatalities and 389846 new cases in 2022, with approximately 120,200 cases (31%) attributed to the use of smokeless tobacco and areca nut products, primarily in regions such as South-Central Asia, Southeast Asia, and Melanesia [1]. Statistical data vary between different countries as well as between different socioeconomic groups, reflecting disparities [2]. However, according to the GBD databases from 1990 to 2021, there is a significant global increase in oral cancer incidence, mortality, and DALYs [3].

Oral squamous cell carcinoma (OSCC) accounts for over 90% of OCs, with incidence typically increasing after the fifth decade of life and peaking in older adults [1-3]. Within Europe, oral cancer represents a significant burden, with more than 65,000 new cases and 25,000 deaths annually across EU countries [1].

Nevertheless, OC is a “preventable disease, where smoking and alcohol-considered major risk factors, are present in 90% of cases, having a synergic effect” [4].

The oral cavity is a potentially accessible site for examination, a situation which increases the possibility for early detection and consequently improves survival rates [3,5]. Worldwide, OC has one of the lowest overall five-year survival rates, close to 50%. Overall poorer survival rate is related to a delay in early diagnosis [3,6]. Patient delay can largely be attributed to a lack of public awareness of the signs and symptoms of the disease [7,8].

In Cyprus, 1070 new cases of OC were reported for the period 1998-2021 [9]. Public Dental Services consider OC as an important public health problem and therefore, in the last 10 years, a strategy was developed with the aim of prevention but also early diagnosis of OC. Among others, the strategy includes the following: a) education of dentists but also other health care professionals, especially general practitioners and nurses working in the community for early identification and prevention of OC, b) publication of information leaflets about OC, c) incorporate information about OC into all oral health education activities, and d) every year, the first week of the month December is dedicated to activities to raise awareness among the public about OC, including free examination to the public, participation of experts in TV-panels, press releases, etc.

The aim of this study was to evaluate the effectiveness of this strategy and more specifically to evaluate whether there was an improvement in the knowledge and perceptions of Cypriots with regard to OC, compared with a similar study that was conducted in 2012. Within this frame, findings will help to decide whether there is a need for modification of the existing strategy.

## Materials and methods

**Figure 1:**
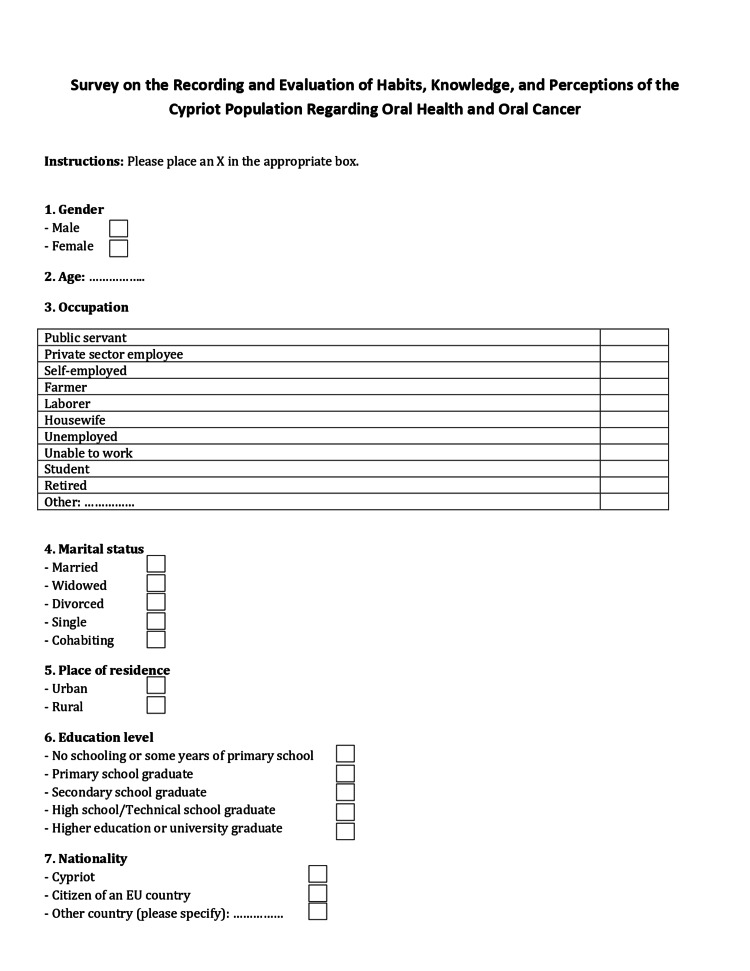
Page 1 of the questionnaire, covering demographic characteristics including gender, age, occupation, marital status, place of residence, education level, and nationality.

**Figure 2:**
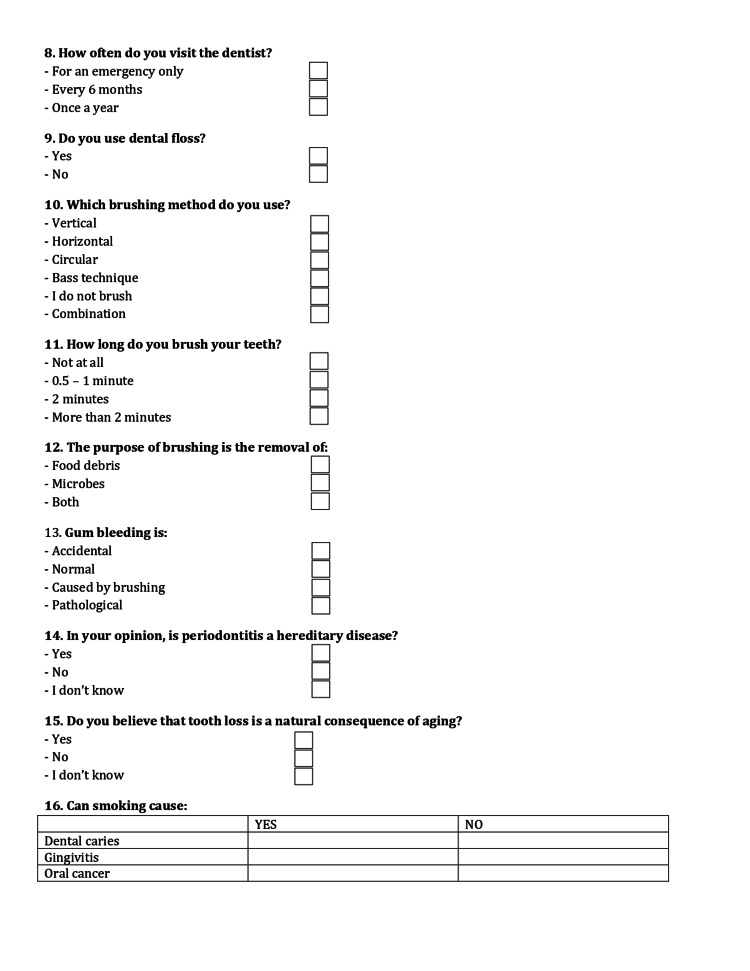
Page 2 of the questionnaire, addressing oral hygiene behaviors and knowledge, such as frequency of dental visits, use of dental floss, toothbrushing methods, duration and purpose of brushing, perceptions of gum bleeding, awareness of periodontitis, and beliefs regarding tooth loss and smoking.

**Figure 3:**
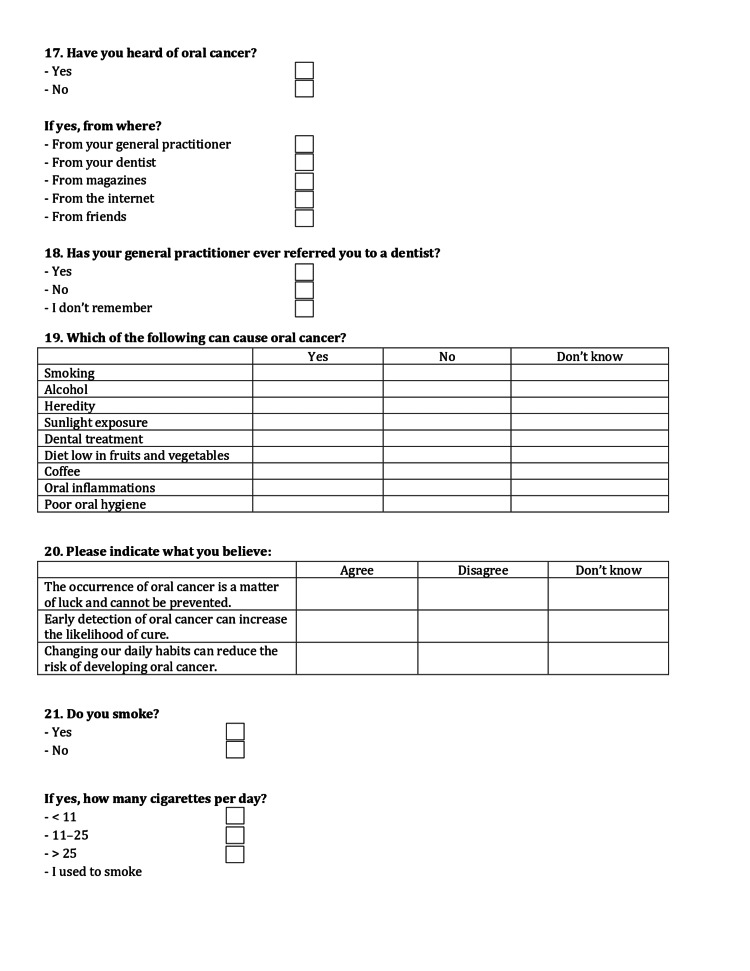
Page 3 of the questionnaire, focusing on awareness of oral cancer, sources of information, physician referral patterns, knowledge of risk factors, and beliefs about prevention and early detection.

**Figure 4:**
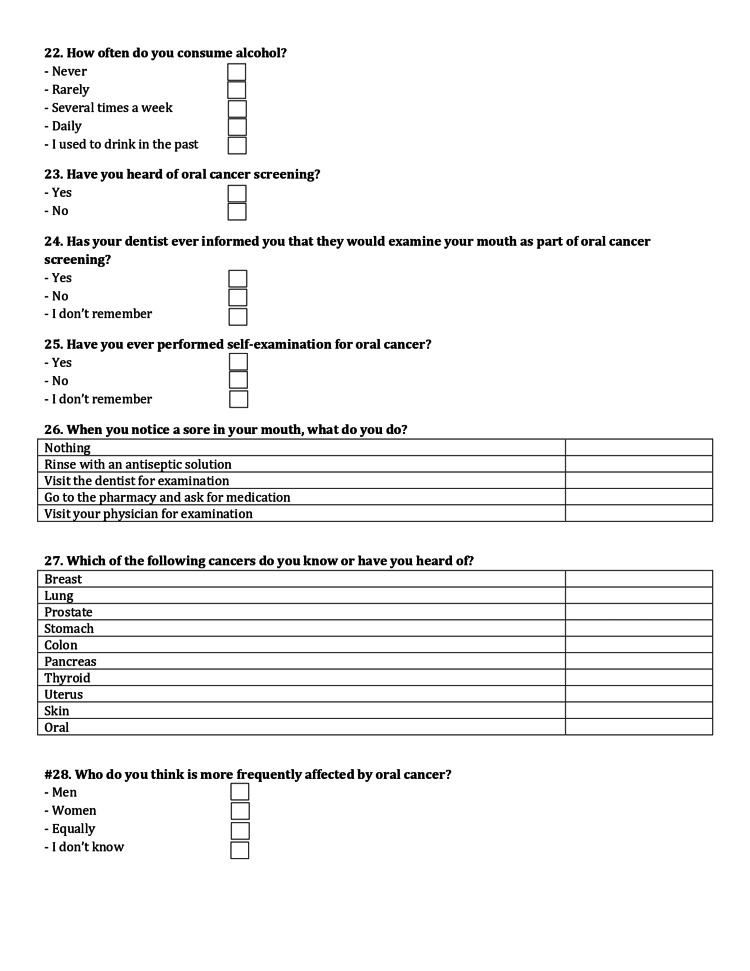
Page 4 of the questionnaire, exploring lifestyle and preventive practices, including smoking and alcohol consumption, awareness and experience with oral cancer screening, self-examination practices, responses to oral lesions, and knowledge of other cancer types.

**Figure 5:**
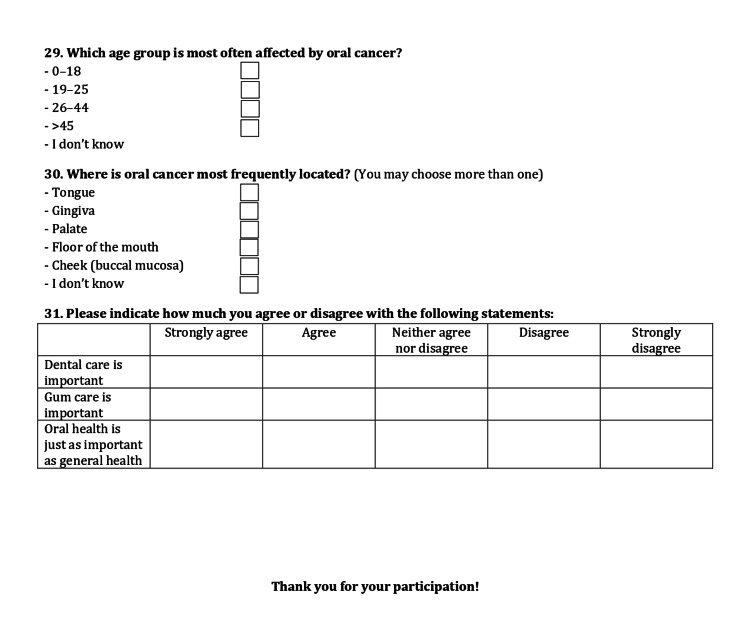
Page 5 of the questionnaire, assessing perceptions about populations most affected by oral cancer (gender and age), common anatomical sites, and attitudes toward the importance of oral and general health.

The study was conducted as part of a preventive campaign for OC, which was organized by the Public Dental Services (PDS) of the Cyprus Ministry of Health and the Clinic of the Oral and Maxillofacial Surgery of the Nicosia General Hospital (NGH) in December 2021. The study met the ethical norms and standards stated in the Declaration of Helsinki and was approved by the National Bioethics Committee of Cyprus (Approval number: 2021.01.178). The campaign was carried out by dentists working for the PDS, who were responsible for several mobile dental clinics at central points in the four major cities of Cyprus. The campaign also included free examinations for those who participated. Prior to the oral examination, participants were asked to fill in an anonymous questionnaire (Figures 1-5), which was the same as the one that was used in a similar study in 2012 [10] and was designed by the clinical staff of the PDS and the Clinic of the Oral and Maxillofacial Surgery of the NGH. Permission to use the questionnaire (not open access) was obtained from the original authors and the relevant authorities.

Data collection was conducted on-site during the national awareness campaign by trained staff of the Public Dental Services, under the supervision of a Public Health Specialist at the Ministry of Health, serving as Coordinator of the Preventive Sector of the Public Dental Services, who also acted as the study’s principal investigator. Participants were informed of the voluntary nature of the study and provided verbal consent prior to completing the questionnaire; those who declined participation were excluded, and no personal identifiers were collected.

Inclusion criteria were being ≥18 years old and having sufficient knowledge of the Greek language, as the questionnaire was only available in Greek.

No formal sample size calculation was performed, as the study employed convenience sampling of all individuals who attended the nationwide oral cancer awareness campaign during the study period.

The face validity of the questionnaire including appropriateness, logical sequence and comprehensibility of the questions was examined by six subjects (four dentists and two patients), while content validity was assessed using the content validity ratio (CVR) and content validity index (CVI) with the help of three experts in oral medicine. The final questionnaire included 21 questions related with socio-economic characteristics, habits such as smoking, alcohol consumption frequency and reason for visiting a dentist, questions about patients’ knowledge regarding the etiology and the epidemiological characteristic of oral cancer, and the source of their information and also questions about their perceptions whether oral cancer can be prevented by changing personal habits as well as whether their dentist has performed a screening examination for oral cancer. Finally, there was a question about “What do you do in case you detect some kind of soreness in your mouth?” A sample of 172 people from the four major cities in Cyprus (Nicosia, Limassol, Larnaca, and Paphos), who visited the four mobile dental clinics, participated in the study.

The statistical analysis of data was carried out using IBM SPSS Statistics for Windows, Version 25 (Released 2017; IBM Corp., Armonk, New York, United States). For categorical variables, the chi-squared (χ²) test was employed to assess associations, while Fisher’s exact test was used for 2×2 contingency tables. A p-value < 0.05 was considered statistically significant. Variables showing significant associations were subsequently included in a multiple logistic regression model. Forward stepwise algorithms were used, with the rejection of those variables that did not fit the model significantly. Odds ratios (OR) and 95% confidence intervals (CIs) were also calculated.

## Results

Table 1 presents socio-demographic characteristics of the participants and their distribution by province, sex, marital status, nationality, age, level of education and area of living (urban or rural) and compares them with the respectively data in 2012. Both samples have similar characteristics which enables comparisons (Table 1).

**Table 1:** Socio-demographic characteristics of participants and comparison with 2012 sample. Values are presented as n (%). Differences were assessed using the chi-squared test (χ²); p<0.05 was considered statistically significant.

		N (2021)	% 2021	% 2012
Province	Nicosia	50	29	32
	Limassol	54	31	21
	Larnaca	48	28	21
	Paphos	20	12	26
Sex	Men	93	54	55
	Women	79	46	44
	Not replied	-	-	1
Age	18-44	32	19	38
	45-64	78	46	41
	65-74	38	26	16
	75+	19	4	4
	Not replied	-	-	1
Marital status	Married	130	76	65
	Single	14	8	17
	Divorced	17	10	8
	Widower	5	3	6
	In a relationship	4	2	3
	Not replied	2	1	1
Area of residence	Urban	134	78	79
	Rural	33	19	20
	Not replied	5	3	1
Level of education	Elementary	26	15	12
	Junior high school graduates	31	18	18
	High school graduates	48	28	24
	Higher education graduates	65	38	42
	Not replied	1	1	4
Citizenship	Cypriot	147	86	86
	EU Country Citizen	13	8	7
	Other	5	3	4
	Not replied	7	3	3

Table 2 presents the habitual routine of the participants as well as their knowledge regarding OC, its risk factors and epidemiological characteristics and compares them with that of the participants in 2012.

**Table 2:** Habits and knowledge regarding oral cancer and comparison with 2012 sample. Values are presented as n (%). Statistical comparisons were performed using the chi-squared test (χ²). OC: Oral cancer p<0.05 was considered statistically significant.

		N (2021)	% 2021	% 2012
Frequency of visiting a dentist	Emergent situation	78	45.3	44.4
	Every 6 months	38	22.1	20.9
	More than 1 year	45	26.2	29.5
	Not replied	11	6.4	5.2
Smoking	Yes	48	28.0	33.8
	No	114	66.0	66.2
	Not replied	10	6.0	
Alcohol consumption	Never	44	26.0	25.0
	Rarely	68	40.0	38.1
	Few times a week	33	19.0	22,6
	Every day	8	5.0	4,3
	Not replied	19	10.0	10.0
Have you ever heard about oral cancer	Yes	127	74 .0(*)	53.0
	No	36	21.0	47.0
	Not replied	10	5.0	
Source of information about OC	General practitioner	13	10.0	17.0
	Dentist	31	25.0 (**)	20.0
	Magazines	21	17.0	17.0
	Internet	36	28.0 (***)	18.5
	Friends	26	20.0	12.0
Smoking as a risk factor for OC		147	86 (****)	77.4
Alcohol as a risk factor for OC		98	57.0	59.4
Solar irradiation as a risk factor for OC		58	34.0	38.9
OC is hereditary		47	27.3	38.9
Frequently occurs in men		38	22.0	24.0
Frequently occurs in people>40		61	36.0	36.0
Tongue is the most common site for OC		114	66.3 (*)	29.0
(*) p<0.1%, (**) 5%				

The main reason for visiting a dentist was for an emergency situation (45.3%, n=78), a percentage which is about the same as in 2012 (44.4%, χ² test, p>0.05).

People living in urban areas were more likely to visit a dentist every six months (25.4%) compared with those in rural areas (9.1%, χ² test, p=0.034). Similarly, participants with higher levels of education (university students) (24.2%) were more likely to attend compared with those who had only elementary education (15.9%, χ² test, p=0.02).

In total, 28% of participants reported being current smokers, while 19% reported consuming alcohol a few times per week. Men were more likely to smoke compared with women (37.9% vs 20%, χ² test, p=0.013), as were people aged 18-44 compared with those aged 65-74 (48.1% vs 22.9%, χ² test, p=0.007).

With regard to their knowledge about OC (Table 2), 74% (n=127) were familiar with the concept of OC. This percentage was statistically higher compared with that reported in 2012 (74% vs 53%, p<0.1%). Participants with higher educational levels reported more often that they had heard about oral cancer compared with those with lower levels of education (83.1% vs 62.5%, χ² test, p=0.05). The main source of information about oral cancer in 2019 was the internet (28%) followed by the dentist (25%) and friends (20%) showing a change in the order compared with that in 2012 which was 20% dentist, 18.5% internet, 17% general medical practitioner and magazines.

As far as risk factors, 86% recognized smoking as the main risk factor, showing a significant increase compared with that in 2012 (77.4%, χ² test, p<0.05). One out of two participants (57%) mentioned alcohol consumption as a risk factor for oral cancer and only 34% solar irradiation.

Regarding knowledge about the epidemiological characteristics of oral cancer, 66.3% recognized the tongue as the most common site, compared with 29% in 2012 (χ² test, p<0.001). Participants visiting the dentist every six months (55.3%) and those with higher education (41%) were more likely to know this compared with those attending only for emergencies (24.4%, χ² test, p=0.003) and those with only elementary education (15.6%, χ² test, p=0.018).

Although almost eight out of ten (74%) were familiar with OC, only 58.7% (n=101) knew about screening for OC, and even fewer reported having undergone a preventive examination by their dentist (19.8%, n=34) or carried out a self-examination (20.3%, n=35). However, these percentages were significantly higher compared with that in 2012 (χ² test, p<0.001; Table 3). People who visited the dentist frequently were more likely to have been screened for oral cancer (33.7%, n=26) and to have carried out a self-examination (30.4%, n=24) compared with those visiting only for emergencies (10.4% and 11.8%, respectively; χ² test, p=0.01).

**Table 3:** Knowledge and perceptions regarding oral cancer prevention and early detection. Values are presented as n (%). Statistical comparisons were performed using the chi-squared test (χ²). OC: Oral cancer p<0.05 was considered statistically significant.

		2021 (%)	2012 (%)
Have you heard of oral cancer?		74.0(*)	53.0
Have you heard about screening for oral cancer?		58.7 (*)	32.5
Have you ever had screening for oral cancer?		19.8 (*)	12.0
Oral cancer is a matter of luck		25.0	25.6
Early detection increases the chances of treatment		73.3	70.5
Changing habits decreases the chance of having OC		64.0	68.4
What you do in case you detect some kind of soreness in your mouth	Go to a dentist	36.6 (**)	25.6
	Use mouthwash	32.0	31.6
	Do nothing	20.4	15.0
	Go to a doctor	8.7	8.5
	Go to a pharmacy	8.1	3.8
(*)p<0.1% (**) 5%			

Regarding the knowledge and perceptions of the participants about prevention and early diagnosis of OC, one out of four participants (25% n=43) mentioned that mouth cancer is a matter of luck, and we cannot prevent it. 64% (n=110) recognized that by changing everyday habits we can reduce the risk for developing OC. 73.3% (n=126) believed that early detection can increase the chances for treatment (Table 3).

Regarding the last question “What you do in case you detect some kind of soreness in your mouth?” 36.6% (n=63) mentioned that they go to a dentist, 32% (n=55) they use mouthwash, 20.4% (n=35), they do nothing, 8.7% (n=15) they visit a doctor and 8.1% (n=14) they go to the pharmacy. The respectively percentages for 2012 were 25.6%, 31.6%, 15%, 8.5% and 3.8% with more people in 2019 choosing to visit a dentist (5%<p<1%).

More men (25.8%, n=24) than women (12.7%, n=10) reported doing nothing when they detected a sore in their mouth (χ² test, p=0.059).

## Discussion

This study confirmed the results of previous studies showing that Cypriots are mainly visiting a dentist for an emergency [11,12]. Infrequent visits to the dentist prevent the promotion of oral health, but also prevent an early screening examination for OC and may delay early diagnosis.

Our findings showed that regularity of visiting the dentist was associated with increased knowledge of OC. Therefore, the importance of regular dental visits for oral examinations and consultation should be emphasized to the public. Also, any barriers that prevent accessibility to the health care system should be removed. Unfortunately, serious disparities in access to health care and to oral health services exist across Europe, especially among low-income populations. According to Eurostat, in 2023, across the entire EU, 4.7% of people aged 16 and over reported unmet needs for dental examination or treatment. Of these unmet needs, 2.9% were attributed specifically to cost. This accounts for roughly three-fifths of all reasons for unmet dental care. Critically, in Romania, Italy, and Spain, at least four in five people with an unmet need for dental care said cost was the reason [13].

The study also revealed gender and age differences regarding daily smoking. National data continue to demonstrate clear gender disparities in daily smoking prevalence, with current estimates in Cyprus indicating that approximately 33% of men and 24% of women use tobacco, which is a substantial decrease in women compared to earlier figures [14]. Although specific age-group prevalence data are limited, smoking remains most common among individuals aged 25-39 (35%) and 40-54 (33%), aligning generally with the previous observation that younger adults smoke more than older groups [15].

Having in mind that the risk for developing OC is increased after the age of 40 years [3], this study highlighted the need for more smoking cessation as well as alcohol use reduction programs among younger people, starting from childhood and continuing during adolescence.

Overall, between 2012 and 2021, there was an improvement in the level of knowledge among people living in Cyprus about OC, as more people reported that they had heard about OC [10]. The increased percentage of people mentioned that they knew about OC can be partly attributed to the increased efforts made by the Dental Services of the Ministry of Health, the Clinic of the Oral and Maxillofacial Surgery of the Nicosia General Hospital and generally the efforts by the dental society and the awareness campaigns that have been organized every year for the last 10 years. However, 25% still said that they had not heard of OC. This gives cause for concern as low levels of awareness will affect the chances for early diagnosis and treatment [3,16].

The main source of information for the participants was the internet (28%), which emphasizes the importance of using public media to inform society about important health issues, including OC. The second source of information was the dentist (25%). Although it has recorded a significant increase compared with the previous study in 2012 [10], it is obvious that dentists should be encouraged to perform their pivotal role in informing the public about risk factors and signs of OC. In addition, continuous educational programs should focus on updating dentists’ knowledge and clinical skills in OC screening.

With regard to the main risk factors, the majority (86%) recognized smoking as the main risk factor, demonstrating a significant increase compared with 2012 (77.4%). This may reflect global tobacco control efforts, which have increased awareness of the danger of tobacco use in recent years [17]. The results of two studies [18,19] have shown that 85-95% of people knew tobacco to be a risk factor, while two other studies [20,21] have found lower percentages (40- 44% -76%). On the other hand, the fact that 28% of the studied population reported current smoking status shows that there are people who continue to smoke, although they know the harmful consequences of smoking, and that information is not always enough to change harmful habits. A population-based survey of adults in the UK has shown that a combination of public education on symptoms and empowerment to seek medical advice, as well as support at the primary level, could enhance early presentation and improve cancer outcomes [22]. Similarly, the “Find Cancer Early” community education campaign in regional Western Australia demonstrated that raising awareness of cancer signs and symptoms and encouraging timely help-seeking can improve the likelihood of earlier presentation and better outcomes [23].

However, as in other studies [24,25], knowledge surrounding other associated risk factors was less extensive. Especially regarding alcohol, which has a synergistic effect with smoking, only 57% answered positively, showing that targeting information about risk factors and their synergism is highly desirable.

The present study showed associations between certain socioeconomic characteristics and knowledge about OC. It was observed that people with higher education levels had better awareness of OC, a result that is consistent with similar previous studies [18,19,21] as well as with a study that was conducted in 2012 [10]. This information is very important as it shows that lower socioeconomic status is associated with an increased risk for OC [3]. Indeed, in a report published by the WHO, the two-way relationship between health and educational level is very well documented [26]. This implies that broader strategies are needed to tackle the social determinants of health.

In agreement with the findings in the UK [27] and an Australian study [19], a low proportion of participants in the current study were aware that they had been checked for OC by their dentist. This is a drawback that must be faced. Using the opportunity provided by dental appointments to raise awareness may be increasingly vital to encouraging early detection of OC. On the other hand, and taking into consideration the infrequent dental visits of Cypriots, opportunistic OC examination undertaken by a health care professional (not only a dentist) may be important in this view. Dental services are not affordable for low-income patients, and thus it is more likely high-risk population, as those coming from lower socioeconomic status, to visit a physician rather than a dentist. In these patients, an opportunistic oral examination should be performed during medical examination. Additionally, training of primary health workers in screening and provision of first-level care in OC is strongly suggested [28]. Therefore, a network of private and public dentists, other healthcare professionals, including General Practitioners, nurses, and health visitors, would form an ideal OC screening team providing access for everyone.

In the present study, although most of the subjects agreed that the detection of OC in early stages can increase the success of the treatment, approximately one out of four believed that developing OC is a matter of luck and, therefore, unavoidable. This may be associated with a fatalism concept also observed in other studies [20], which can be a critical obstacle to changing lifestyle.

Patient delay remains a critical barrier to early diagnosis in OC, often resulting from misinterpretation of symptoms or reliance on self-management. In the present study, most participants reported engaging in some form of self-treatment, such as using mouth rinses or choosing to wait and observe whether their symptoms would resolve without professional intervention. This behavior aligns with findings from a Southeast England study, where the median delay in presentation among young people with OC was five weeks [29], and nearly one-third of individuals with potentially malignant lesions waited over three months before seeking care [7]. Despite such trends, encouraging data from the Cyprus National Cancer Registry indicate that localized disease (Stage I) comprised the largest proportion of diagnosed cases, with 38% of OC cases detected at an early stage. Advanced-stage diagnoses involving regional or distant spread were comparatively lower, with Stage III and IV cases totaling 29%, and distant metastases reported in only 6% of cases. These data may reflect some degree of public awareness or timely clinical response, but the self-reported hesitation to seek early evaluation underscores the persistent need for targeted public education campaigns and professional vigilance to further reduce diagnostic delay [30].

Strengths and limitations

Some limitations to this study should be considered in interpreting its results. One of the limitations was the data collection method, which was completely self-reported; hence, the ability to validate the findings is limited.

In addition, the pool of respondents came from a specific group of people who participated in the cancer awareness campaign. It would have been ideal to survey a random sample of the general population, but resources were limited. The relatively modest sample size further restricts external validity, and caution is required when generalizing the findings of this survey to the Cypriot population. 

However, it is emphasized that the same methodology was followed in both periods, the same questionnaire was distributed, and the demographic characteristics of the study population were the same as those who participated in the previous study; therefore, comparisons could be considered reliable. 

Furthermore, it should be noted that the survey was conducted during the COVID-19 pandemic, which influenced the number of people who visited the mobile dental clinics. However, even under these difficult circumstances, the Cyprus Ministry of Health did not downplay the importance of OC and successfully carried out the campaign.

This study also did not set out to determine whether dentists are performing full and thorough screening. It is possible that screening may indeed be taking place, but dentists may not be communicating this fact to their patients.

## Conclusions

Overall, the data support that knowledge among people in Cyprus regarding OC has improved over the last 10 years, although there are still inequalities among different socioeconomic groups. On the other hand, the percentage of those who answered that have been screened for OC, although it has been improved, is still poor, and it seems that there is a significant loss of valuable time from the time they notice a lesion in the oral cavity until they visit a dentist or a doctor. Based on the findings of the study, the strategy of the Cyprus Ministry of Health for the prevention and early detection of OC is considered successful and is moving in the right direction. However, reducing both patient delay for early detection and professional diagnostic delay remains an ongoing target. Therefore, improving patient awareness with clear messages that will prompt those with symptoms to consult a health professional is of vital importance to shorten the appraisal interval. On the other hand, protocols should be designed to include opportunistic oral examinations in both dental and medical practices. For that purpose, additional training is required for all the professionals who will increase their confidence and consistency in undertaking oral cancer examinations and in talking with their patients about tobacco and alcohol use.

Lastly, since low socioeconomic status is a risk factor for OC, attempts to address this health inequity should occur at the government level and focus on improved access and affordability to oral health care. A dedicated oral health budget would be ideal to ensure appropriate funding and facilitate the integration of oral health care into Universal Health Coverage packages in line with the WHO Global Oral Health Strategy.
